# Biomechanical Strategies to Improve Running: Cadence, Footwear, and Orthoses—A Quasi-Experimental Study

**DOI:** 10.3390/s25082414

**Published:** 2025-04-11

**Authors:** Yves Lescure, Marie Adelaide Nicolas, Eleonore Perrin, Enrique Sanchis-Sales, Eva Lopezosa-Reca, Corentin Travouillon, Gabriel Gijon-Nogueron

**Affiliations:** 1 Facultad Ciencias de la Salud, Universidad de Malaga, 29071 Malaga, Spain; ma-nicolaspeyrot@ecole-rockefeller.com (M.A.N.); evalopezosa@uma.es (E.L.-R.); gagijon@uma.es (G.G.-N.); 2 Department of Podologie, l’Ecole Rockefeller, 69008 Lyon, France; eleonore-perrin@ecole-rockefeller.com; 3 Facultad de Enfermería y Podología, Universidad de Valencia, 46010 Valencia, Spain; ensansa@uv.es; 4 TRINOMA Co., 48800 Villefort, France; corentin.travouillon@gmail.com

**Keywords:** running, biomechanics, cadence, foot orthoses, kinematic analysis

## Abstract

**Background:** Running-related injuries are often associated with biomechanical inefficiencies, particularly in the sagittal and frontal planes. This study evaluates the effects of three interventions—reduced heel-to-toe drop (HTD) shoes, increased cadence, and inversion foot orthoses—on key kinematic parameters: ankle dorsiflexion, knee flexion, and hip adduction (measured at foot strike and at their respective peak joint angles during the stance phase). **Methods:** Nineteen recreational runners (ten males and nine females; mean ± SD: age 26.4 ± 4.3 years; height 174.2 ± 7.8 cm; weight 68.3 ± 9.6 kg; BMI 22.5 ± 2.1 kg/m^2^) participated in a 3D motion capture study under five experimental conditions: baseline (10 mm HTD, no cadence adjustment, no foot orthoses), full intervention (5 mm HTD, +10% cadence, orthoses), and three partial interventions: HTD combined with orthoses, HTD combined with increased cadence, and cadence increase alone. Kinematic changes were analyzed for statistical significance. **Results:** The full intervention significantly increased ankle dorsiflexion at foot strike (from 8.11° to 10.44°; *p* = 0.005) and reduced peak knee flexion (from 45.43° to 43.07°; *p* = 0.003). Cadence adjustments consistently produced improvements, while orthoses and HTD alone showed effects on ankle flexion only. **Conclusions:** Combining structural (HTD and orthoses) and dynamic (cadence) modifications optimizes running biomechanics, providing evidence-based strategies for injury prevention and performance enhancement.

## 1. Introduction

Running is one of the most widely practiced physical activities worldwide, offering substantial health benefits such as improved cardiovascular fitness, enhanced mental well-being, and a reduced risk of chronic diseases [1,2,3]. However, it is also associated with a high prevalence of overuse injuries, with annual incidence rates reported between 19% and 79% among recreational runners [4,5,6]. Overuse injuries [5] typically result from repetitive mechanical loading that exceeds the body’s ability to adapt and recover, often affecting the lower limbs [7,8].

Some of the most common running-related injuries include patellofemoral pain syndrome (PFP) [6]. This condition is characterized by anterior knee pain, often linked to elevated patellofemoral joint (PFJ) stress due to excessive kinematic lower limb values likes peak knee flexion during midstance [9] and peak hip adduction [10,11]. PFP is one of the most frequent injuries among runners, particularly those with biomechanical inefficiencies such as excessive hip adduction, contralateral pelvic drop, or high peak knee flexion during stance phase [12,13,14].

Iliotibial band syndrome (ITBS) is considered the second most common knee pain in runners, after patellofemoral pain syndrome, and accounts for approximately one-tenth of all running injuries [15]. Associated with lateral knee pain, ITBS often arises from excessive hip adduction and poor frontal plane stability, leading to increased strain on the iliotibial band [10,16,17]. Finally, tibial stress fractures are also very common [18]. These injuries are linked to repetitive impact forces during the ground contact phase, particularly in runners who exhibit overstriding or high braking forces [19], which is linked to the foot and ankle position at foot strike.

The high prevalence of these injuries underscores the multifactorial etiologies and the need of multimodal management [20] including running load management, muscular reinforcement [20,21,22] and targeted biomechanical interventions that are aimed at optimizing running mechanics to minimize injury risk while maintaining performance [23].

Indeed, biomechanics play a central role in running-related injuries, with inefficiencies in the sagittal and frontal planes being key contributors to overuse injuries. These inefficiencies may concern ankle, knee, and hip kinematics, which collectively influence lower limb biomechanics [24]. For example, ankle biomechanics is known to have a key role in the load distribution on the kinetic chain. Furthermore, in the sagittal plane, excessive knee flexion during midstance increases PFJ stress, compressive loads, and quadriceps activation, elevating the risk of PFP. Runners who exhibit high knee flexion often experience reduced shock absorption efficiency and increased anterior knee pain [25,26]. In the frontal plane, excessive hip adduction reflects poor pelvic stability, often accompanied with contralateral pelvic drop (CPD). These factors increase lateral knee strain and contribute to conditions such as ITBS and medial collapse syndromes [27]. Poor frontal plane control may also increase ground reaction force misalignment, exacerbating strain on the lower extremities [28,29].

Several biomechanical interventions have been proposed to address these inefficiencies and reduce injury risk. These include modifying running cadence, altering footwear geometry, and incorporating orthotic devices [30,31,32,33].

Cadence adjustments involve increasing the number of steps per minute, typically by 5–10%. This reduces stride length, lowers vertical oscillation, and decreases braking forces during the ground contact phase [34,35]. Increased cadence has been shown to significantly reduce peak knee flexion and PFJ stress, making it an effective strategy for managing PFP [36,37]. It also indirectly improves frontal plane stability by minimizing CPD and excessive hip adduction [38].

Moreover, running shoes with a lower reduced heel-to-toe drop (HTD) encourage midfoot or forefoot striking, redistributing mechanical loads from the knee to the ankle. This shift reduces knee extensor moments, braking forces, and vertical loading rates, offering a potential solution for runners prone to PFP and tibial stress injuries [19,26].

Finally, in runners, foot orthoses (FOs) are mainly utilized to treat and/or prevent overuse injuries and improve running performance. Even if the evidence supporting their effectiveness is mixed, it seems that foot orthoses can redistribute loads to uninjured structures, resulting in immediate pain relief and potentially aiding in injury treatment [39]. Plus, customized foot orthoses, are frequently employed to mitigate the biomechanical irregularities [32]. They can enhance foot mechanics and optimize muscle activation in the lower extremities, leading to an amelioration of PFPS symptoms. Expert consensus on patellofemoral pain advises the short-term application of foot orthoses to alleviate pain [40,41].

Despite previous studies examining each intervention performance and consequences in isolation [38,40,42], there is limited evidence regarding their combined biomechanical effects on recreational runners. Therefore, this study aims to address this gap by evaluating some combined effects of cadence adjustment, footwear modifications, and foot orthoses on lower limb kinematics.

We hypothesize that the combination of increased cadence, reduced heel-to-toe drop, and inversion foot orthoses will lead to significant improvements in running biomechanics, particularly on biomechanical parameters like ankle and knee flexion and hip adduction at different events of the running stride foot strike, peak timing, and time series analysis. By integrating findings from previous research with new experimental data, this study seeks to provide evidence-based recommendations for clinicians and sports scientists working to optimize running mechanics and reduce injury risks.

## 2. Materials and Methods

### 2.1. Protocol and Registration

This study conforms to all STROBE guidelines and reports the required information accordingly. The study was carried out in full accordance with the Declaration of Helsinki on ethical principles for medical research involving human subjects, and was approved by the Ethics Committee of the University of Malaga (CEUMA 206-2023-H), Spain.

### 2.2. Participants

The original study group was composed of 19 healthy runners. The participants included 10 males and 9 females with a mean age of 26.4 ± 4.3 years, a height of 174.2 ± 7.8 cm, a weight of 68.3 ± 9.6 kg, and a body mass index (BMI) of 22.5 ± 2.1 kg/m^2^ (Table 1).

The study data were obtained from February to November 2024 and recruitment occurred at Ecole Rockefeller (Lyon, France). All subjects were at least 18 years old and were able to follow the study instructions.

Participants were recreational runners with at least one year of consistent training (minimum two sessions per week), and none reported a history of major lower limb injuries or surgeries within the previous 12 months [42]. This was verified through a structured pre-screening questionnaire to control for potential confounding factors.

The inclusion criteria were as follows: (I) age between at least 18 years and 45 years [18,23]; (II) recreationally active runners (at least 2 running sessions per week) who were able to run on a treadmill at a speed of 10–12 km/h for six minutes [23]; and (III) rearfoot striking runners wearing neutral shoes with 10 mm heel-to-toe drop.

The exclusion criteria were as follows: degenerative bone and joint disease (diagnosed from medical history); lower limb surgery; recent knee/ankle injuries or serious foot injury that could have left morphological alterations; painful cutaneous conditions such as callus or plantar warts.

After receiving detailed information on the objectives and procedures of the study, each subject signed an informed consent form to participate.

### 2.3. Material

Three-dimensional motion capture was used to assess kinematic changes.

Biomechanical data were collected using a Qualisys Motion Capture System (TRINOMA, Villefort, France) with eight Miqus M3 cameras (2 Mpx resolution, operating at 340 Hz). This system is widely recognized for its high temporal and spatial accuracy, making it ideal for biomechanical research on dynamic activities such as running. The Qualisys 17-Marker Lower Limb Gait Model was used to analyze sagittal and frontal plane kinematics. This setup comprises 17 reflective markers (14 mm) placed on key anatomical landmarks:

- (I) Pelvis Markers: Bilateral anterior superior iliac spines (ASIS) and sacrum.
- (II) Thigh Markers: Lateral femoral condyle, anterior tibial tuberosity, and a technical marker positioned above the patella.
- (III) Shank Markers: Lateral malleolus only, placed directly on the skin for precise tracking.
- (IV) Foot Markers: Second and fifth metatarsal heads (placed on the shoes) and calcaneus (placed on the shoes).

This marker set adheres to standardized protocols developed for use with the Qualisys Motion Capture System, ensuring the reliable and accurate measurement of lower-limb joint kinematics during treadmill running trials.

A high-resolution treadmill was used for controlled-speed running trials.

While the Qualisys Motion Capture System is appropriate for this research, the selection of ankle dorsiflexion, knee flexion, and hip adduction as kinematic variables is justified by their strong association with common running-related injuries. Increased peak knee flexion angle and peak hip adduction angle have been linked to patellofemoral pain [10,11,12,14,43] and iliotibial band syndrome [16,17]. Peak hip adduction has also been linked to tibial stress fracture by some authors [18,44] while ankle dorsiflexion influences shock absorption and stride pattern, all of which are modifiable through biomechanical interventions [6,8,9,10,11,12,27].

**Figure 1:**
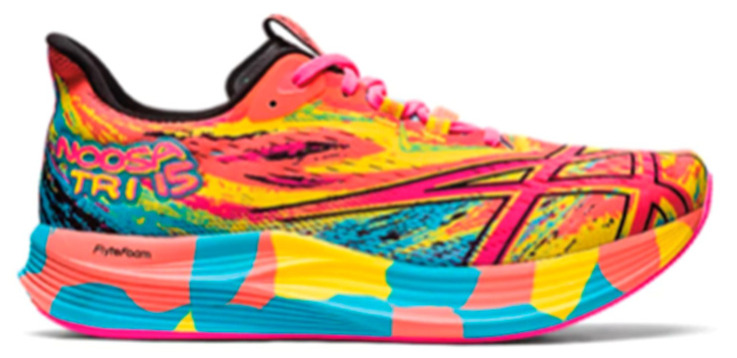
Profile view of the Noosa model from Asics.

The running shoes used were ASICS NOOSA (Figure 1) (characteristics: heel-to-toe drop 5 mm; weight: 255 g; men’s size 9). Midsole Technology: FlyteFoam^®^ (Onitsuka Co., Ltd., Tokyo, Japan) for lightweight cushioning, incorporating organic fibers for enhanced durability. Outsole Design: Wet Grip Rubber, engineered for high traction on wet surfaces. Upper: No-sew, breathable mesh for optimal comfort.

#### Foot Orthoses

**Figure 2:**
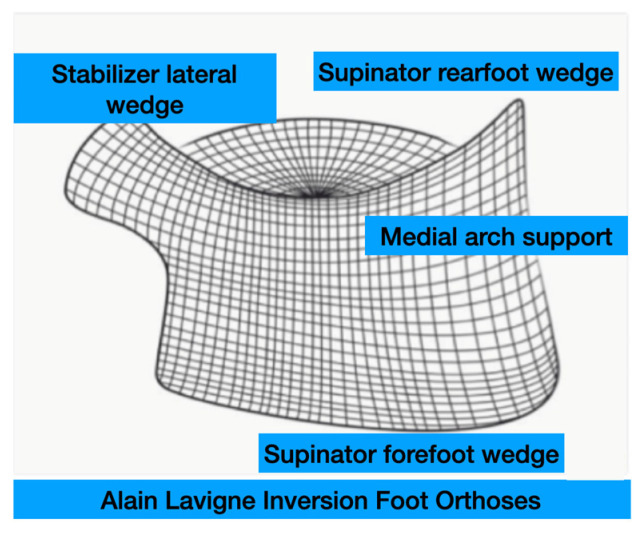
Front view of Alain Lavigne Inversion Foot Orthoses design.

The foot orthoses used were a thermoformed “Alain Lavigne Inversion Foot Orthoses” (ALIFOrthoses) [45] with a full-length medial wedge and a Shore A hardness of 35. The foot orthoses were composed of a “supinating rearfoot wedge”, a medial arch support, a “supinated forefoot wedge”, and a “stabilizer lateral wedge” (Figure 2).

### 2.4. Design

This was a quasi-experimental study including a repeated measure design with one study population. Each participant was randomly evaluated across under five conditions:

C1 (Baseline): Participants ran in their own shoes with a 10 mm heel-to-toe drop, no cadence adjustment, and no orthoses. The decision to allow the use of each participant’s own shoes (with consistent 10 mm HTD) was intended to replicate real-life running conditions and enhance ecological validity. Additionally, all selected shoes were neutral running models with similar cushioning properties to limit variability.

C2 (Full Intervention): Asics Noosa shoes with a 5 mm heel-to-toe drop, cadence increased by 10%, and Alain Lavigne Inversion Foot Orthoses (ALIFOrthoses).

C3 (HTD + Orthoses): Asics Noosa shoes with ALIFOrthoses only.

C4 (HTD + Cadence): Asics Noosa shoes with a 10% cadence increase, no orthoses.

C5 (Cadence Only): Participants’ own shoes with a 10 mm heel-to-toe drop and a 10% cadence increase.

The five conditions were selected to isolate and combine the biomechanical effects of cadence increase, reduced HTD, and inversion foot orthoses. The “HTD only” condition was not included to limit participant fatigue and avoid redundancy, as previous studies have already analyzed its isolated effects [42,46]. Instead, we focused on practical combinations relevant for clinical and performance settings.

The order of the five running conditions was randomized for each participant using a computer-generated randomization list, ensuring that no condition consistently preceded or followed another. This reduced order effects and participant adaptation bias.

Before initiating the experimental conditions, participants completed a five-minute warm-up on the treadmill at a self-selected speed while wearing their own shoes. This warm-up ensured participants were comfortable and had reached a steady running state, reducing potential variability during the trials.

To minimize the influence of fatigue as a confounding factor, participants were provided with rest intervals of at least five minutes between conditions. During this time, they were seated, hydrated, and instructed to avoid strenuous activity.

Participants ran on a treadmill at a controlled speed of 12 km/h. Each condition consisted of two minutes of running, with the first minute designated for familiarization and the second minute for data collection to ensure steady-state kinematics. Sufficient rest was provided between trials to avoid fatigue.

Although treadmill running offers controlled environmental conditions ideal for motion capture, it may differ slightly from overground running in stride mechanics and ground reaction patterns. However, previous studies have shown that kinematic differences between treadmill and overground running are minimal in recreational runners at moderate speeds, supporting the generalizability of our findings [47].

### 2.5. Outcome Measures

The primary kinematic outcomes were as follows:

1. Ankle Dorsiflexion (AF): Measured at foot strike (FS) and peak timing (MAX).
2. Knee Flexion (KF): Measured at FS and peak timing.
3. Hip Adduction (HA): Measured at FS and peak timing.

The curves of these three movements over the average running cycle will be analyzed and compared under each condition using the Root Mean Square Deviation. This global statistical measure will allow us to quantify the difference between two curves over the entire running cycle [48].

### 2.6. Sample Size

For the three conditions, the required sample size was calculated using a 95% confidence level and 80% power. This power level ensures a reasonable probability of detecting a significant difference if it truly exists. The effect size was determined based on the smallest observed difference between the means of the conditions, using the pooled standard deviation. The sample size analysis indicated that a minimum of 19 participants would be sufficient to meet these statistical requirements, based on preliminary data from a pilot analysis of 6 recreational runners.

### 2.7. Statistical Analysis

Marker trajectories were processed using Qualisys Track Manager software (2024.3) to compute joint angles and evaluate kinematic differences across conditions. Dunnett’s test was used to compare each intervention condition (C2–C5) with the control (C1), with a threshold set at *p* < 0.05. All the analyses were conducted using MATLAB (R2021a, 8.3.0.532, The MathWorks Inc., Natick, MA, USA).

Root Mean Square Deviation (RMSD) was computed for each running cycle to compare the effects of the different interventions according to the moment in the cycle [48].

## 3. Results

**Figure 3:**
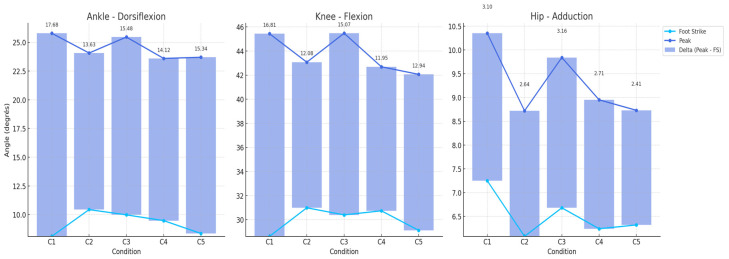
Graphical representation of joint angle changes across all conditions.

The results highlight the acute biomechanical effects of the five experimental conditions on peak knee flexion and peak hip adduction. Detailed comparisons are provided below for each comparison of conditions. All the conditions are compared to C1 (baseline condition) (Figure 3).

### 3.1. Ankle Dorsiflexion (AF)

#### 3.1.1. At Foot Strike (FS): (Figure 4)

***Condition C2 (Full Intervention) compared to C1 (Baseline):***

Participants exhibited an average ankle dorsiflexion angle of 8.1055 at foot strike, representing the baseline performance in their own shoes (10 mm heel-to-toe drop) and average ankle dorsiflexion angle of 10.44 at foot strike with the full intervention (5 mm heel-to-toe drop, +10% cadence, and ALIFOrthoses), which mean a significant increase in ankle dorsiflexion angle at an FS of 2.33° (*p* = 0.005).

**Figure 4:**
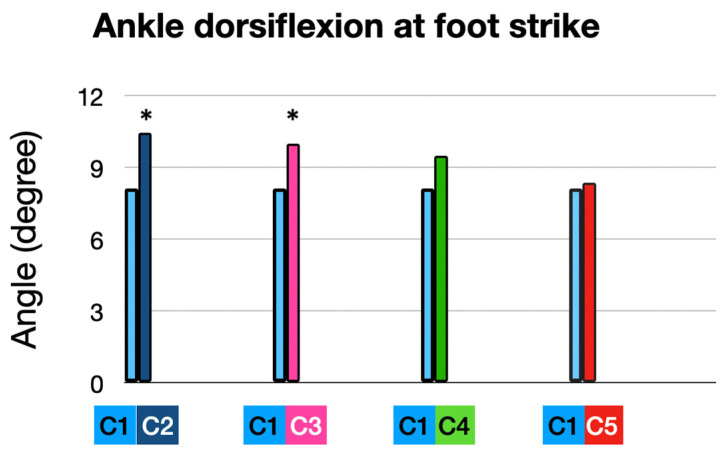
Ankle dorsiflexion at foot strike: comparison of interventions to baseline condition. * means significant *p* value < 0.05.

***Condition C3 (HTD + ALIFOrthoses) compared to C1:***

When runners used the Asics Noosa (5 mm heel-to-toe drop) combined with ALIFOrthoses, this led to a significant increase in ankle dorsiflexion angle of 1.87° (from 8.11 to 9.98° (*p* = 0.036)).

***Condition C4 (HTD + Cadence) compared to C1:***

Adding a 10% cadence increase to the Asics Noosa produced a trend toward an increased ankle dorsiflexion of 1.38°, from 8.10° to 9.48°, but this was statistically non-significant (*p* = 0.17).

***Condition C5 (Cadence Only) compared to C1:***

Cadence adjustment alone, in the participants’ own shoes, produced no change.

#### 3.1.2. At Peak Timing (MAX): (Figure 5 and Figure 6)

***Condition C2 (Full Intervention) compared to C1 (Baseline):***

Participants exhibited an average peak ankle dorsiflexion angle of 25.80°, representing the baseline performance in their own shoes (10 mm heel-to-toe drop), and an average peak dorsiflexion angle of 24.07° with the full intervention (5 mm heel-to-toe drop, +10% cadence, and ALIFOrthoses), which mean a trend toward a decrease in peak ankle dorsi-flexion of 1.73° (*p* = 0.096).

**Figure 5:**
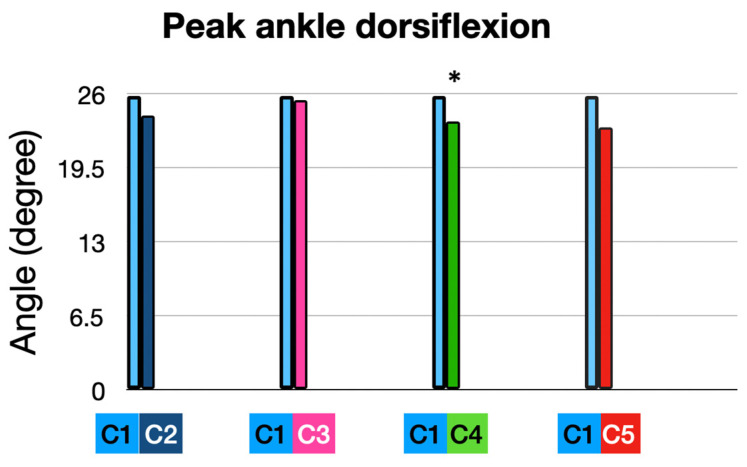
Peak ankle dorsiflexion: comparison of interventions to baseline condition. * means significant *p* value < 0.05.

**Figure 6:**
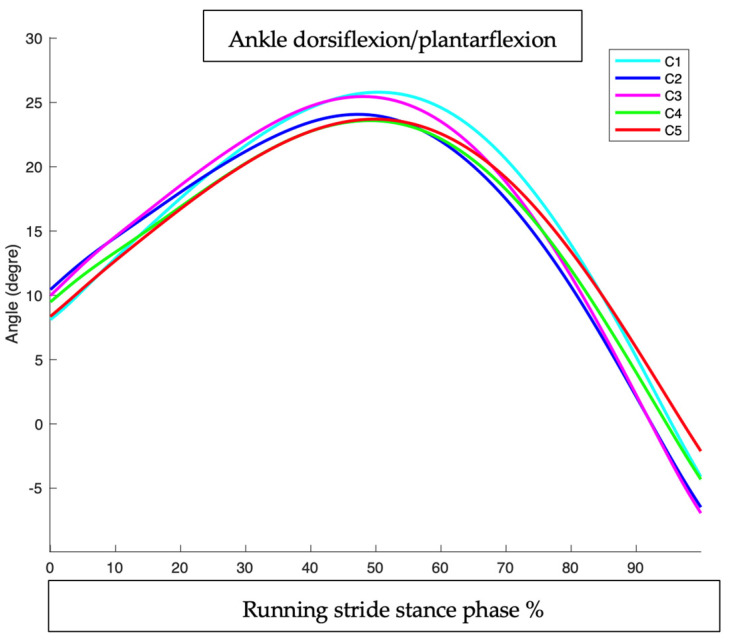
Ankle dorsiflexion/plantarflexion during running stride stance phase in all conditions.

***Condition C3 (HTD + ALIFOrthoses) compared to C1:***

When runners used the Asics Noosa (5 mm heel-to-toe drop) combined with ALI-FOrthoses, we observed no change in peak ankle dorsiflexion.

***Condition C4 (HTD + Cadence) compared to C1:***

Adding a 10% cadence increase to the Asics Noosa reduced peak ankle dorsiflexion from 25.80° to 23.59° (−2.21°; *p* = 0.032), showing the importance of dynamic interventions like cadence.

***Condition C5 (Cadence Only) compared to C1:***

Cadence adjustment alone, in the participants’ own shoes, seemed to achieve a reduction in ankle dorsiflexion from 25.80° to 23.70° (−2.10°; *p* = 0.07), showing the role of cadence in reducing sagittal plane joint angles.

#### 3.1.3. Root Mean Square Deviation (RMSD) (Table 2)

***Condition C2 (Full Intervention) compared to C1 (Baseline):***

This intervention showed the most pronounced effect at the end of the cycle (after 50%). This suggests an influence on the push-off phase.

**Table 2:** Ankle dorsiflexion (RMSD, in degrees) during the stance phase of the running stride: comparison of interventions to baseline condition.

Stride Cycle	C1/C2	C1/C3	C1/C4	C1/C5
**0–10%**	2.1938	1.9377	1.1584	0.1894
**10–20%**	1.1205	1.369	0.3508	0.5252
**20–30%**	0.2697	0.7646	1.0402	1.1168
**30–40%**	0.7926	0.3316	1.6005	1.6131
**40–50%**	1.4316	1.1692	2.0218	1.9715
**50–60%**	2.1906	0.7145	2.3309	2.1029
**60–70%**	2.8769	1.4178	2.4349	1.8182
**70–80%**	3.186	2.0816	2.1884	1.0881
**80–90%**	3.2148	2.7118	1.6716	0.3553
**90–100%**	2.8176	2.9954	0.8049	1.3843

***Condition C3 (HTD + ALIFOrthoses) compared to C1:***

This intervention had a moderate effect throughout the cycle but remained weaker than C2.

***Condition C4 (HTD + Cadence) compared to C1:***

This intervention showed a progressive effect, with the maximum impact occurring between 40% and 70% of the cycle.

***Condition C5 (Cadence Only) compared to C1:***

This intervention had a moderate effect at the beginning of the cycle, but its impact was weak by the end of the cycle.

At the beginning of the cycle (0–30%), the effect of the interventions was minimal.

The maximum impact was observed between 50% and 80% of the cycle, corresponding to the push-off. After 80%, only C1/C2 and C1/C3 maintained a notable effect on ankle flexion (Table 2).

### 3.2. Knee Flexion (KF)

#### 3.2.1. At Foot Strike (FS) (Table 3 and Figure 7 and Figure 8)

***Condition C2 (Full Intervention) compared to C1 (Baseline):***

Participants exhibited a knee flexion angle of 28.62° at foot strike, representing the baseline performance in their own shoes (10 mm heel-to-toe drop), and a knee flexion angle of 30.99° at foot strike with the full intervention (5 mm heel-to-toe drop, +10% cadence, and ALIFOrthoses), which mean a significant increase at an FS of 2.37° (*p* = 0.0255).

***Condition C3 (HTD + ALIFOrthoses) compared to C1:***

When runners used the Asics Noosa (5 mm heel-to-toe drop) combined with ALIFOrthoses, this led to a trend toward an increase in knee flexion angle at FS (from 28.62° to 30.40°; *p* = 0.13).

***Condition C4 (HTD + Cadence) compared to C1:***

Adding a 10% cadence increase to the Asics Noosa produced a significant increase in knee flexion angle at FS of 2.12° from 28.62° to 30.74° (*p* = 0.05).

***Condition C5 (Cadence Only) compared to C1:***

Cadence adjustment alone, in the participants’ own shoes, produced no change.

**Table 3:** Biomechanical outcomes: comparison of all interventions to baseline condition. (*) means significant *p* value < 0.05.

Biomechanical Criteria	Mean 1st Condition C1	Condition	Mean 2nd Condition	Delta	*p*-Value	Cohen’s d
**Ankle dorsiflexion at foot strike**	8.11°	C2	10.44°	2.33°	0.0055 *	0.94
		C3	9.98	1.87°	0.0360 *	0.75
		C4	9.48°	1.37°	0.1790	0.55
		C5	8.36°	0.26°	0.99	0.10
**Peak ankle dorsiflexion**	25.79°	C2	24.07°	−1.72°	0.0962	−0.88
		C3	25.46°	−0.33°	0.923	−0.84
		C4	23.6°	−2.19°	0.0316 *	0.96
		C5	23.70	−2.09	0.0714	0.76
**Knee flexion at foot strike**	28.62°	C2	30.99°	2.37°	0.0255 *	0.85
		C3	30.40°	1.88°	0.13	0.20
		C4	30.74°	2.12°	0.05 *	−0.95
		C5	29.12°	0.5°	0.9	−1.10
**Peak knee flexion**	45.43°	C2	43.07°	−2.36°	0.0318 *	−1.36
		C3	45.47°		1	−0.66
		C4	42.69°	−2.74°	0.0066 *	−0.65
		C5	42.06°	−3.37	0.001 *	0.94
**Hip adduction at foot strike**	7.25°	C2	6.08°	−1.17°	0.15	0.75
		C3	6.68°	−0.67°	0.72	0.55
		C4	6.24°	−1.01°	0.25	0.10
		C5	6.32°	−0.95°	0.32	−0.88
**Peak hip adduction**	10.35°	C2	8.72°	−1.63	0.15	−0.84
		C3	9.84°	−0.51	0.9	0.96
		C4	8.95°	−1.40°	0.23	0.76
		C5	8.73°	−1.62	0.16	0.85

**Figure 7:**
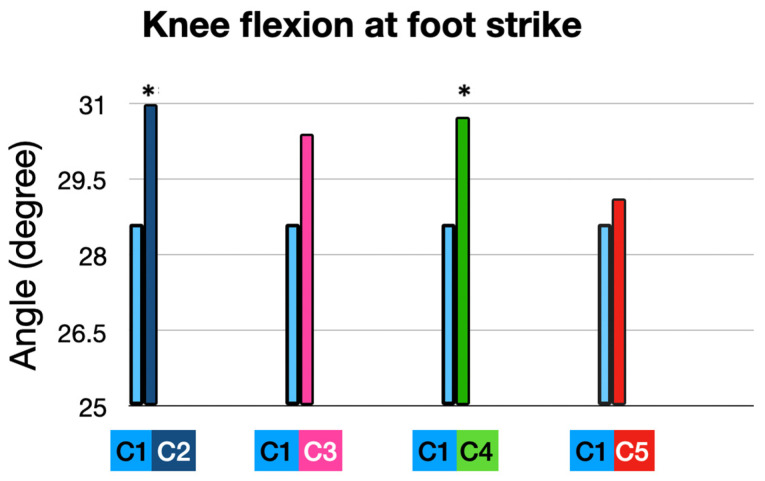
Knee flexion at foot strike: comparison of interventions to baseline condition. (*) means significant *p* value < 0.05.

**Figure 8:**
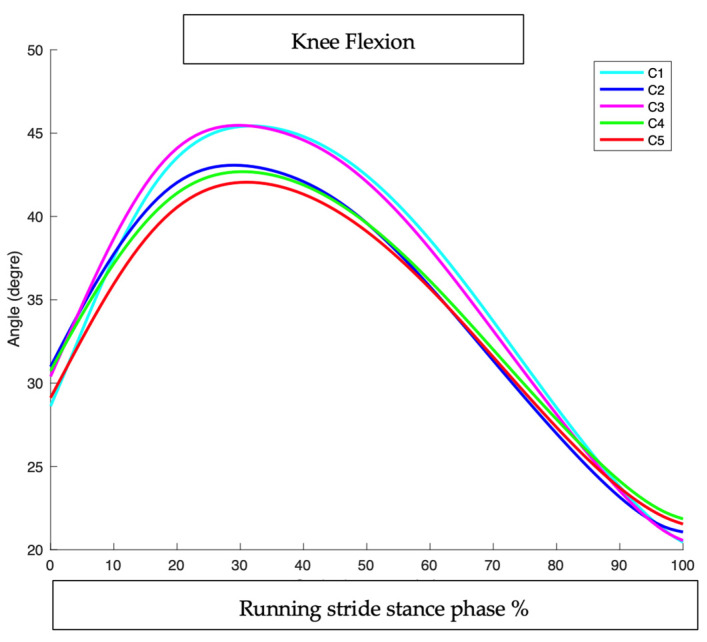
Knee flexion during running stride stance phase in all conditions.

#### 3.2.2. At Peak Timing (MAX): (Figure 8 and Figure 9 and Table 3)

***Condition C2 (Full Intervention) compared to C1 (Baseline):***

Participants exhibited an average peak knee flexion angle of 45.43°, representing the baseline performance in their own shoes (10 mm heel-to-toe drop), and an average peak knee flexion angle of 43.07° with the full intervention (5 mm heel-to-toe drop, +10% cadence, and ALIFOrthoses), which mean a significant decrease in knee flexion of 2.36° (*p* = 0.003).

**Figure 9:**
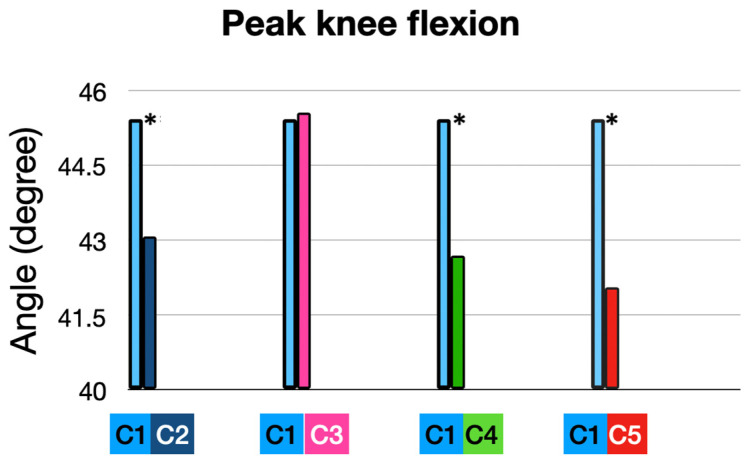
Peak knee flexion: comparison of interventions to baseline condition. (*) means significant *p* value < 0.05.

***Condition C3 (HTD + ALIFOrthoses) compared to C1:***

When runners used the Asics Noosa (5 mm heel-to-toe drop) combined with ALIFOrthoses, the peak knee flexion did not change.

***Condition C4 (HTD + Cadence) compared to C1:***

Adding a 10% cadence increase to the Asics Noosa reduced knee flexion significantly from 45.43 to 42.69° (−2.74°; *p* = 0.0066), showing the importance of dynamic interventions like cadence.

***Condition C5 (Cadence Only) compared to C1:***

Cadence adjustment alone, in the participants’ own shoes, achieved a similar reduction in knee flexion from 45.43° to 42.05° (−3.37°; *p* < 0.001), emphasizing the dominant role of cadence in reducing sagittal plane loading (Figure 6 and Figure 7).

#### 3.2.3. Root Mean Square Deviation (RMSD) (Table 4)

***Condition C2 (Full Intervention) compared to C1 (Baseline):***

This intervention significantly modified knee flexion by 2.04°.

**Table 4:** Knee flexion (RMSD, in degrees) during the stance phase of the running stride: comparison of interventions to baseline condition.

Stride Cycle	C1/C2	C1/C3	C1/C4	C1/C5
**0–10%**	1.6	1.55	1.28	0.73
**10–20%**	0.82	0.88	1.39	2.35
**20–30%**	1.93	0.37	2.45	3.19
**30–40%**	2.53	0.12	2.82	3.41
**40–50%**	2.78	0.28	2.91	3.44
**50–60%**	2.86	0.46	2.72	3.19
**60–70%**	2.65	0.57	2.16	2.6
**70–80%**	2.03	0.5	1.29	1.75
**80–90%**	1.18	0.33	0.38	0.76
**90–100%**	0.37	0.16	0.87	0.58

***Condition C3 (HTD + ALIFOrthoses) compared to C1:***

This intervention had minimal influence on knee kinematics (0.66°).

***Condition C4 (HTD + Cadence) compared to C1:***

This intervention produced a decrease of 2.01°, indicating that cadence is a key factor in modifying knee flexion.

***Condition C5 (Cadence Only) compared to C1:***

Cadence alone had a greater impact than the full intervention, with a decrease of 2.45°.

### 3.3. Hip Adduction

#### 3.3.1. At Foot Strike (FS):

None of the conditions produced statistically significant changes in hip adduction at foot strike compared to the baseline.

**Figure 10:**
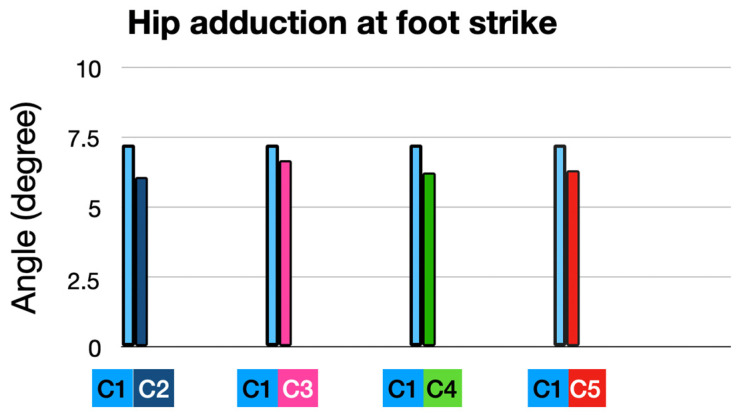
Hip adduction at foot strike: comparison of interventions to baseline condition.

The greatest trend toward reductions were observed in the Full Intervention (C2), indicating that a combination of HTD reduction, cadence increase, and orthoses may be the most effective approach (Figure 10).

#### 3.3.2. At Peak Timing (MAX): (Table 3 and Figure 11 and Figure 12)

None of the conditions produced statistically significant changes in hip adduction at foot strike compared to the baseline.

**Figure 11:**
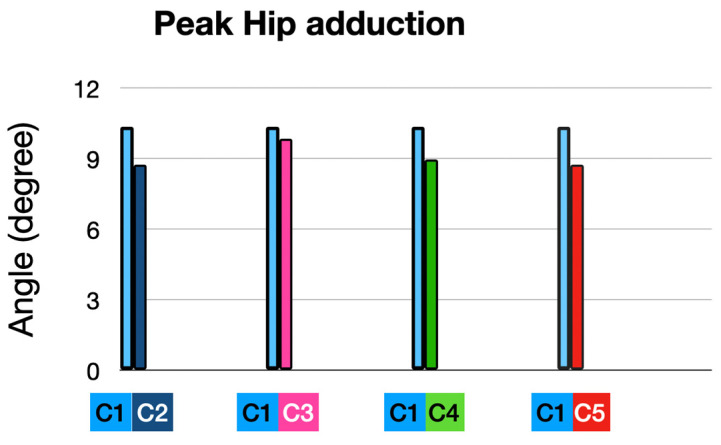
Peak hip adduction: comparison of interventions to baseline condition.

**Figure 12:**
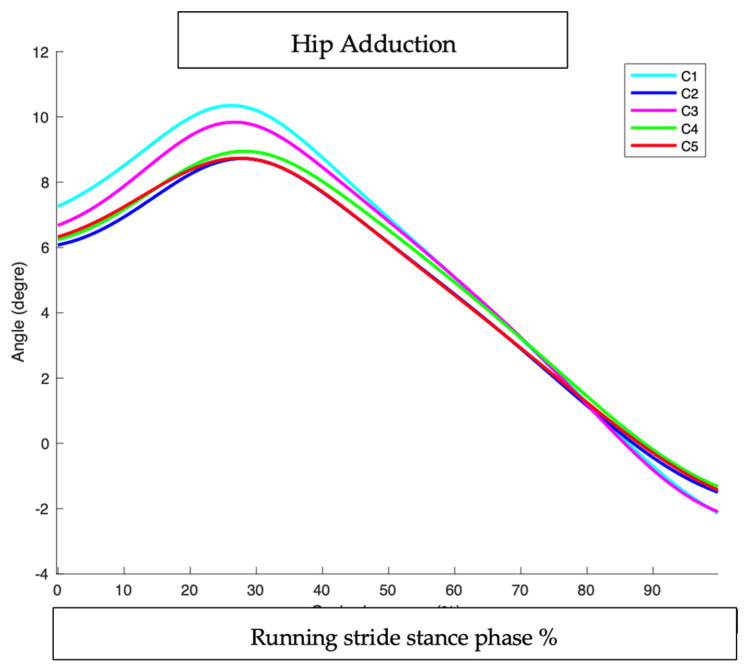
Hip adduction during running stride stance phase in all conditions.

The greatest trend toward reductions were observed in the full intervention (C2), indicating that a combination of HTD reduction, cadence increase, and orthoses may be the most effective approach.

#### 3.3.3. Root Mean Square Deviation (RMSD) (Table 5)

The full intervention, C2, slightly reduced hip adduction (1.04°), but the effect was minimal. We can observe that the largest decrease occurred around the peak timing.

**Table 5:** Hip adduction (RMSD, in degrees) during the stance phase of the running stride: comparison of interventions to baseline condition.

Stride Cycle	C1/C2	C1/C3	C1/C4	C1/C5
**0–10%**	1.3833	0.6213	1.1824	1.0823
**10–20%**	1.6662	0.5977	1.4336	1.4257
**20–30%**	1.672	0.5256	1.4542	1.6167
**30–40%**	1.3083	0.4008	1.0377	1.3048
**40–50%**	0.9155	0.212	0.5601	0.9082
**50–60%**	0.6492	0.054	0.2692	0.6661
**60–70%**	0.425	0.0252	0.0872	0.4381
**70–80%**	0.2313	0.0237	0.1026	0.19
**80–90%**	0.1225	0.0901	0.3461	0.223
**90–100%**	0.4546	0.0757	0.6606	0.567

The cadence-only intervention, C5, appeared to play a role in reducing hip adduction with the same timing although the impact was not substantial.

## 4. Discussion

The results of our study support our initial hypothesis: the combined intervention—including a reduction in heel-to-toe drop, an increase in cadence, and the implementation of Alain Lavigne foot orthoses—leads to meaningful changes in running biomechanics. Among all tested conditions, this multimodal strategy appears to be the most effective in optimizing kinematic parameters, particularly at the knee and ankle levels.

### 4.1. Impact of Full Interventions (C2: HTD + Cadence + ALIFOrthoses)

The full intervention (C2) combined reduced heel-to-toe drop (HTD), increased cadence, and inversion foot orthoses. By integrating dynamic (cadence adjustments) and structural (HTD and orthoses) modifications, this intervention aimed to optimize sagittal and frontal plane kinematics more effectively than the other interventions.

#### 4.1.1. Effects of C2 on the Ankle

C2 significantly increased ankle dorsiflexion at a foot strike (FS) of 2.33° (*p* = 0.005).

As our participants were all rearfoot strikers, reduced HTD encouraged a tendency toward a midfoot strike pattern, shifting the center of mass forward and increasing ankle dorsiflexion [46]. This increase in dorsiflexion promoted a more anterior foot strike pattern, enhancing shock absorption and reducing vertical loading rates, and may reduce the risk of TSF [19].

C2 also produced a non-significant trend toward a decrease in peak ankle dorsiflexion compared to the baseline (C1) (−1.72°; *p* = 0.096; Cohen’s d = −0.88).

This decreased tendency, confirmed by the Root Mean Square Deviation analysis, seems to have the most pronounced effect at the end of the cycle (after 50%). This seems to be mainly due to the increased cadence [49] which is also known to shorten step length, reducing ground contact time but also the rearfoot maximum force, and may reduce the risk of TSF too [30].

Furthermore, it seems that HTD and ALIFOrthoses are more linked to the impact at foot strike while cadence adjustments seem more linked to a decrease in peak dorsiflexion [49].

#### 4.1.2. Effects of C2 on Knee Flexion (KF)

C2 significantly increased knee flexion at FS (2.37°; *p* = 0.0255) reducing patellofemoral joint (PFJ) stress. In fact, a randomized controlled trial by Roper and al. demonstrated that increased knee flexion at IC was linked to the decrease in PFPS symptoms [50].

Additionally, peak knee flexion was significantly reduced, indicating improved impact attenuation and quadriceps efficiency [42]. The Root Mean Square Deviation confirms this conclusion, showing that the decrease in knee flexion due to C2 continued during the pushing phase of the stride.

Therefore, these combined effects decreased quadriceps demand, reducing PFJ loading and enhancing overall shock attenuation.

Shoes with a 5 mm heel-to-toe drop have been shown to promote midfoot or forefoot strike patterns, potentially reducing sagittal plane loading on the knee joint [42,51]. This aligns with the observed reduction in peak knee flexion in our study.

Moreover, increasing cadence by 10% has consistently been reported to reduce knee loading during running and to relieve the pain in PFPS runners [37,38,52].

The use of inversion foot orthoses (ALIFOrthoses) is known to improve knee function [40] and to be useful in the treatment of PFPS runners [40]. It may enhance frontal plane stability by aligning the subtalar joint, indirectly facilitating knee flexion by optimizing frontal plane mechanics [32,33,40].

#### 4.1.3. Effects of C2 on Hip Adduction (HA)

C2 modestly reduced hip adduction throughout the stance phase, enhancing frontal plane stability. Increasing cadence by 10% has consistently been reported to decrease peak hip adduction. Our results are not in line with the literature, suggesting two options:

- The addition of HTD and ALIFOorthoses is harmful and decreases the impact of increasing step rate on the hip kinematics.
- Our study recruited healthy runners whereas most studies showing good results on peak hip adduction were on PFPS runners with a greater peak hip adduction at baseline. Both Neal’s [38] and Bramah’s [37] study with increased cadence intervention showed an average peak hip adduction at baseline of around 15°. In our study, the average at baseline was only 10.35. This low peak adduction in healthy runners may explain the lack of significance of C2 on hip adduction.

In conclusion, the full intervention (C2) effectively enhanced ankle dorsiflexion and knee flexion, with a modest non-significant reduction in hip adduction, supporting its potential application in PFP, ITBS, and TSF management. Specifically, it has been described that increased peak knee flexion angle during the stance phase of running appears to be a biomechanical factor associated with PFP, especially for male recreational runners. C2 appears to be a way to alter this parameter [12].

### 4.2. Impact of Structural Modifications Alone (C3: HTD + ALIFOrthoses)

#### 4.2.1. Effects of C3 on the Ankle

C3 significantly increased ankle dorsiflexion at a foot strike (FS) of 1.87° (*p* = 0.036). The influence of ALIFOrthoses on this kinematic criterion is supported by another study [53]. Comparing the effect of C3 to that of C4 on the ankle kinematics allows us to highlight that ALIFOrthoses contributes to a better effect at foot strike than the increased cadence of 10% combined with HTD in C4.

#### 4.2.2. Effects of C3 on Knee Flexion and Hip Adduction (KF)

It should be noted that C3 led to a non-significant trend toward an increase in knee flexion angle at FS (from 28.62° to 30.40°; *p* = 0.13), suggesting an interesting trend toward PFPS runner management [32,40,54].

Nevertheless, the lack of significant changes in both peak knee flexion and hip adduction in C3 indicates that structural modifications alone seem insufficient to produce meaningful biomechanical improvements, especially in lower limb kinematics. This is in line with a recent systematic review that showed that foot orthoses control ankle and tibial motion at the coronal and transverse planes, but the effects on hip and knee kinematics were not evident [55].

Conversely, two studies [33,56] investigating runners with patellofemoral pain syndrome (PFPS) demonstrated an immediate alteration in hip kinematics—specifically a reduction in peak hip adduction—when running with foot orthoses (FOs). These findings further support the notion that individuals exhibiting greater baseline peak hip adduction may be more susceptible to the biomechanical influence of FOs. Then, foot orthoses may influence hip kinematics in overpronators or individuals with medial collapse tendencies, but such effects may not be generalized to healthy recreational runners without abnormal loading patterns [54].

Furthermore, other biomechanical evaluation criteria should be explored in the future to really understand the impact of foot orthoses on lower limb biomechanics. For example, researchers have shown that the medial knee compartment load during running was linked to the external knee adduction moment (EKAM) [57] and that the use of lateral wedge insoles decreases both EKAM and the medial knee compartment load [58,59,60]. A study evaluating the knee extension moment during running would be interesting to understand the consequences of ALIFOrthoses on patellofemoral stress [42].

### 4.3. Impact of Condition C4 (HTD + Cadence)

#### 4.3.1. Effects of C4 on the Ankle

C4 was the only intervention to significantly decrease peak angle dorsiflexion. According to the literature, the role of cadence in that combination should be dominant for that outcome [30,61] as, like we already mentioned, increased cadence is known to shorten step length. Surprisingly, increased cadence alone (C5) did not significantly reduce peak angle dorsiflexion but only showed a trend toward a decrease. It seems that, in our study, HTD potentiates the cadence effect on ankle dorsiflexion.

#### 4.3.2. Effects of C4 on the Knee and the Hip

C4 achieved comparable but lesser reductions in knee flexion than the full intervention both at foot strike and at peak, underlining the lack of evidence of the mechanism of the foot orthoses’ impact on the knee [40].

The lack of a result on hip adduction can be explained in the same way as for C2.

### 4.4. Effectiveness of Cadence Adjustment Alone (C5)

Effects of C5 on the Ankle, the Knee, and the Hip

C5 showed a real trend toward decreasing peak angle dorsiflexion (−2.09°; *p* = 0.07) and the greatest and most significant results on peak knee flexion (−3.37°; *p* < 0.001). This highlights that this type of “running retraining” [62] can be an effective tool to treat knee injuries like PFPS [37,38,63]. Secondly, it apparently has no side effects on the ankle (and even some benefits) compared to “from rearfoot strike to forefoot strike” running retraining, which incurs an overload of the Achilles tendon, as described by Sinclair and al. [52].

The lack of a result on hip adduction can be explained in the same way as for C2.

### 4.5. Clinical Implications

Regarding our results, the full intervention (C2) appears to be the best compromise as C2 seems to have a benefit and significant effect on almost all the ankle and knee criteria (foot strike, peak, and RMSD).

These findings are consistent with the current literature and reinforce the importance of addressing running-related injuries through a comprehensive approach. Notably, the systematic review by Alexander et al. [20] which examined 30 randomized controlled trials, highlighted the multifactorial nature of running-related knee injuries and emphasized the need for multimodal intervention strategies [20]. Their work, titled “*Strategies to prevent and manage running-related knee injuries*”, found that no single intervention alone—whether footwear modification, exercise therapy, or educational programs—consistently reduced the risk of knee injury. However, the review did suggest that a running retraining technique, particularly aimed at reducing impact forces through softer landings, may significantly lower injury risk.

Our findings support this perspective. By combining structural modifications (footwear and orthoses) with dynamic adjustments (cadence increase), we observed more robust and consistent improvements in joint mechanics than with any single intervention alone. This suggests that when it comes to the prevention or management of overuse injuries such as patellofemoral pain syndrome or tibial stress fractures, an integrative multi-modal strategy may offer the most clinically meaningful outcomes.

Regarding individualized interventions, the efficiency of cadence adjustments suggests that it should be a primary focus in biomechanical rehabilitation programs. Indeed, the reductions in knee flexion have been associated with a decreased risk of overuse injuries such as patellofemoral pain syndrome (PFPS) and iliotibial band syndrome [9,64]. Several studies and recent work also showed that decreasing knee flexion and hip adduction is linked to a significant biomechanical and clinical improvement on PFPS runners [23,34,37,38,63]. Then, isolated cadence increase (C5) produced improvements in knee mechanics, aligning with previous studies suggesting that increasing step rate reduces stride length and associated braking forces [27,28]. This reinforces the effectiveness of cadence retraining as a low-cost, accessible intervention.

Structural modifications and especially foot orthoses can be considered as adjunctive measures, particularly for runners with specific needs and maybe specific clinical predictors [32,54]. Thus, a recent scoping review evoked that integrating FOs into a comprehensive treatment plan is suggested to yield better results compared to stand-alone first-line treatments [39].

Nonetheless, further research is needed to explore the optimal integration of FOs into injury-specific treatment plans. However, it is noteworthy that foot orthoses may provide additional benefits for specific populations, such as those with existing knee pathologies [41,54].

As for footwear recommendations, the use of lower heel-to-toe drop shoes may be encouraged to promote efficient running mechanics, although individual responses should be evaluated [42,51].

The dominance of cadence adjustments in improving running mechanics associated with the limited efficacy of structural modifications alone emphasizes the importance of dynamic interventions. The potential of combining interventions in C2 to achieve maximal biomechanical optimization seems to be most effective but needs to be confirmed by future studies including clinical follow-up.

### 4.6. Study Limitations

This study has several limitations. First, it involved a small sample of 19 healthy recreational runners, which may limit generalizability to injured populations or elite athletes. Second, the interventions were acute, and no long-term adaptations or clinical outcomes were assessed. Third, allowing participants to wear their own shoes in the baseline condition—although ecologically valid—may have introduced variability in footwear characteristics beyond the heel-to-toe drop.

Future research should investigate the long-term effects of these interventions, particularly in populations with known biomechanical risk factors or prior injuries. Randomized controlled trials with follow-up periods could evaluate whether biomechanical changes translate into lower injury incidence. Additionally, subgroup analyses (e.g., overpronators vs. neutral runners) may help tailor orthotic or cadence-based interventions to individual needs.

Additionally, as evoked previously, only kinematic parameters were analyzed; future studies should incorporate kinetic data (e.g., joint moments and ground reaction forces) to provide a more complete biomechanical profile. Finally, we did not include a condition isolating HTD and FOs alone due to concerns about participant fatigue and redundancy, which might have limited the full separation of effects.

## 5. Conclusions

This study suggests the significant biomechanical benefits of combining a reduced heel-to-toe drop, cadence adjustments, and Alain Lavigne Inversion Foot Orthoses on running kinematics. Specifically, the full intervention significantly alters critical factors in minimizing joint loading and may be a good tool to prevent and manage running-related injuries. Furthers studies are needed to understand the biomechanical mechanisms of foot orthoses. Cadence seems to play a key role in running biomechanics too.

## Figures and Tables

**Table 1:** Participant characteristics.

	Mean	SD
Age/years	26.4	±4.3
Height/cm	174	±7.8
Weight/kg	68.3	±9.6
Body Mass Index	22.5	±2.1

## Data Availability

The data are unavailable due to privacy or ethical restrictions.

## References

[1] Lee D.-C., Pate R.R., Lavie C.J., Sui X., Church T.S., Blair S.N. (2014). Leisure-time running reduces all-cause and cardiovascular mortality risk. J. Am. Coll. Cardiol..

[2] Oja P., Titze S., Kokko S., Kujala U.M., Heinonen A., Kelly P., Koski P., Foster C. (2015). Health benefits of different sport disciplines for adults: Systematic review of observational and intervention studies with meta-analysis. Br. J. Sports Med..

[3] Szabo A., Abrahám J. (2013). The psychological benefits of recreational running: A field study. Psychol. Health Med..

[4] Kakouris N., Yener N., Fong D.T.P. (2021). A systematic review of running-related musculoskeletal injuries in runners. J. Sport Health Sci..

[5] van Mechelen W. (1992). Running injuries. A review of the epidemiological literature. Sports Med. Auckl. NZ.

[6] van Gent R.N., Siem D., van Middelkoop M., van Os A.G., Bierma-Zeinstra S.M.A., Koes B.W. (2007). Incidence and determinants of lower extremity running injuries in long distance runners: A systematic review. Br. J. Sports Med..

[7] Aicale R., Tarantino D., Maffulli N. (2018). Overuse injuries in sport: A comprehensive overview. J. Orthop. Surg..

[8] Edwards W.B. (2018). Modeling Overuse Injuries in Sport as a Mechanical Fatigue Phenomenon. Exerc. Sport Sci. Rev..

[9] Garza-Borjón A., González-González M., de la Garza-Salazar J.F., Simental-Mendía M., Acosta-Olivo C. (2024). Understanding the patho-anatomy of patellofemoral pain: A crucial foundation for comprehensive management. Orthop. Rev..

[10] Noehren B., Hamill J., Davis I. (2013). Prospective evidence for a hip etiology in patellofemoral pain. Med. Sci. Sports Exerc..

[11] Noehren B., Pohl M.B., Sanchez Z., Cunningham T., Lattermann C. (2012). Proximal and distal kinematics in female runners with patellofemoral pain. Clin. Biomech..

[12] Yang C., Best T.M., Liu H., Yu B. (2022). Knee biomechanical factors associated with patellofemoral pain in recreational runners. Knee.

[13] Boling M., Padua D., Marshall S., Guskiewicz K., Pyne S., Beutler A. (2010). Gender differences in the incidence and prevalence of patellofemoral pain syndrome. Scand. J. Med. Sci. Sports.

[14] Neal B.S., Barton C.J., Birn-Jeffery A., Morrissey D. (2019). Increased hip adduction during running is associated with patellofemoral pain and differs between males and females: A case-control study. J. Biomech..

[15] Sanchez-Alvarado A., Bokil C., Cassel M., Engel T. (2024). Effects of conservative treatment strategies for iliotibial band syndrome on pain and function in runners: A systematic review. Front. Sports Act. Living.

[16] Aderem J., Louw Q.A. (2015). Biomechanical risk factors associated with iliotibial band syndrome in runners: A systematic review. BMC Musculoskelet. Disord..

[17] Ceyssens L., Vanelderen R., Barton C., Malliaras P., Dingenen B. (2019). Biomechanical Risk Factors Associated with Running-Related Injuries: A Systematic Review. Sports Med. Auckl. NZ.

[18] Milner C.E., Foch E., Gonzales J.M., Petersen D. (2023). Biomechanics associated with tibial stress fracture in runners: A systematic review and meta-analysis. J. Sport Health Sci..

[19] Daoud A.I., Geissler G.J., Wang F., Saretsky J., Daoud Y.A., Lieberman D.E. (2012). Foot strike and injury rates in endurance runners: A retrospective study. Med. Sci. Sports Exerc..

[20] Alexander J.L.N., Culvenor A.G., Johnston R.R.T., Ezzat A.M., Barton C.J. (2022). Strategies to prevent and manage running-related knee injuries: A systematic review of randomised controlled trials. Br. J. Sports Med..

[21] Dutton R.A., Khadavi M.J., Fredericson M. (2014). Update on Rehabilitation of Patellofemoral Pain. Curr. Sports Med. Rep..

[22] Saltychev M., Dutton R., Laimi K., Beaupré G., Virolainen P., Fredericson M. (2018). Effectiveness of conservative treatment for patellofemoral pain syndrome: A systematic review and meta-analysis. J. Rehabil. Med..

[23] de Souza Júnior J.R., Rabelo P.H.R., Lemos T.V., Esculier J.-F., Barbosa G.M.P., Matheus J.P.C. (2024). Effects of two gait retraining programs on pain, function, and lower limb kinematics in runners with patellofemoral pain: A randomized controlled trial. PLoS ONE.

[24] Willwacher S., Kurz M., Robbin J., Thelen M., Hamill J., Kelly L., Mai P. (2022). Running-Related Biomechanical Risk Factors for Overuse Injuries in Distance Runners: A Systematic Review Considering Injury Specificity and the Potentials for Future Research. Sports Med. Auckl. NZ.

[25] Hryvniak D., Magrum E., Wilder R. (2014). Patellofemoral Pain Syndrome: An Update. Curr. Phys. Med. Rehabil. Rep..

[26] Bonacci J., Vicenzino B., Spratford W., Collins P. (2014). Take your shoes off to reduce patellofemoral joint stress during running. Br. J. Sports Med..

[27] Lashien S.A., Abdelnaeem A.O., Gomaa E.F. (2024). Effect of hip abductors training on pelvic drop and knee valgus in runners with medial tibial stress syndrome: A randomized controlled trial. J. Orthop. Surg..

[28] Räisänen A.M., Pasanen K., Krosshaug T., Vasankari T., Kannus P., Heinonen A., Kujala U.M., Avela J., Perttunen J., Parkkari J. (2018). Association between frontal plane knee control and lower extremity injuries: A prospective study on young team sport athletes. BMJ Open Sport Exerc. Med..

[29] Ford K.R., Myer G.D., Hewett T.E. (2003). Valgus knee motion during landing in high school female and male basketball players. Med. Sci. Sports Exerc..

[30] Anderson L. (2022). What is the Effect of Changing Running Step Rate on Injury, Performance and Biomechanics?. A Systematic Review and Meta-analysis. Sports Med..

[31] Dos Santos A.F., Nakagawa T.H., Lessi G.C., Luz B.C., Matsuo H.T.M., Nakashima G.Y., Maciel C.D., Serrão F.V. (2019). Effects of three gait retraining techniques in runners with patellofemoral pain. Phys. Ther. Sport Off. J. Assoc. Chart. Physiother. Sports Med..

[32] Barton C.J., Munteanu S.E., Menz H.B., Crossley K.M. (2010). The efficacy of foot orthoses in the treatment of individuals with patellofemoral pain syndrome: A systematic review. Sports Med. Auckl. NZ.

[33] Boldt A.R., Willson J.D., Barrios J.A., Kernozek T.W. (2013). Effects of medially wedged foot orthoses on knee and hip joint running mechanics in females with and without patellofemoral pain syndrome. J. Appl. Biomech..

[34] Esculier J.-F., Bouyer L.J., Dubois B., Fremont P., Moore L., McFadyen B., Roy J.-S. (2018). Is combining gait retraining or an exercise programme with education better than education alone in treating runners with patellofemoral pain?A randomised clinical trial. Br. J. Sports Med..

[35] Musgjerd T., Anason J., Rutherford D., Kernozek T.W. (2021). Effect of Increasing Running Cadence on Peak Impact Force in an Outdoor Environment. Int. J. Sports Phys. Ther..

[36] Liu Y., Qi Y., Song Y., Feng L., Wang L. (2023). Influences of altering footstrike pattern and cadence on lower extremity joint coordination and variability among runners with patellofemoral pain. PLoS ONE.

[37] Bramah C., Preece S.J., Gill N., Herrington L. (2019). A 10% Increase in Step Rate Improves Running Kinematics and Clinical Outcomes in Runners With Patellofemoral Pain at 4 Weeks and 3 Months. Am. J. Sports Med..

[38] Neal B.S., Barton C.J., Birn-Jeffrey A., Daley M., Morrissey D. (2018). The effects & mechanisms of increasing running step rate: A feasibility study in a mixed-sex group of runners with patellofemoral pain. Phys. Ther. Sport Off. J. Assoc. Chart. Physiother. Sports Med..

[39] Del Duchetto F., Dussault-Picard C., Gagnon M., Dixon P., Cherni Y. (2024). Can Foot Orthoses Benefit Symptomatic Runners? Mechanistic and Clinical Insights Through a Scoping Review. Sports Med.—Open.

[40] Chen Z., Wu J., Wang X., Ren Z. (2022). The effect of foot orthoses for patients with patellofemoral pain syndrome: A systematic review and meta-analysis. Heliyon.

[41] Crossley K.M., Stefanik J.J., Selfe J., Collins N.J., Davis I.S., Powers C.M., McConnell J., Vicenzino B., Bazett-Jones D.M., Esculier J.-F. (2016). 2016 Patellofemoral pain consensus statement from the 4th International Patellofemoral Pain Research Retreat, Manchester. Part 1: Terminology, definitions, clinical examination, natural history, patellofemoral osteoarthritis and patient-reported outcome measures. Br. J. Sports Med..

[42] Zhang M., Zhou X., Zhang L., Liu H., Yu B. (2022). The effect of heel-to-toe drop of running shoes on patellofemoral joint stress during running. Gait Posture.

[43] Boling M.C., Padua D.A., Marshall S.W., Guskiewicz K., Pyne S., Beutler A. (2009). A prospective investigation of biomechanical risk factors for patellofemoral pain syndrome: The Joint Undertaking to Monitor and Prevent ACL Injury (JUMP-ACL) cohort. Am. J. Sports Med..

[44] Pohl M.B., Mullineaux D.R., Milner C.E., Hamill J., Davis I.S. (2008). Biomechanical predictors of retrospective tibial stress fractures in runners. J. Biomech..

[45] Lavigne A., Lescure Y., Delacroix S. (2017). La podologie orthopédique avancée. Rev. Podol..

[46] Yu P., He Y., Gu Y., Liu Y., Xuan R., Fernandez J. (2022). Acute Effects of Heel-to-Toe Drop and Speed on Running Biomechanics and Strike Pattern in Male Recreational Runners: Application of Statistical Nonparametric Mapping in Lower Limb Biomechanics. Front. Bioeng. Biotechnol..

[47] Van Hooren B., Fuller J.T., Buckley J.D., Miller J.R., Sewell K., Rao G., Barton C., Bishop C., Willy R.W. (2020). Is Motorized Treadmill Running Biomechanically Comparable to Overground Running? A Systematic Review and Meta-Analysis of Cross-Over Studies. Sports Med. Auckl. NZ.

[48] Sekine M., Tamura T., Yoshida M., Suda Y., Kimura Y., Miyoshi H., Kijima Y., Higashi Y., Fujimoto T. (2013). A gait abnormality measure based on root mean square of trunk acceleration. J. Neuroengineering Rehabil..

[49] Farina K.A., Hahn M.E. (2021). Increasing Step Rate Affects Rearfoot Kinematics and Ground Reaction Forces during Running. Biology.

[50] Roper J.L., Doerfler D., Kravitz L., Dufek J.S., Mermier C. (2017). Gait Retraining From Rearfoot Strike to Forefoot Strike does not change Running Economy. Int. J. Sports Med..

[51] Zhang M., Shi H., Liu H., Zhou X. (2021). Biomechanical Analysis of Running in Shoes with Different Heel-to-Toe Drops. Appl. Sci..

[52] Sinclair J.K. (2016). Effects of a 10 week footstrike transition in habitual rearfoot runners with patellofemoral pain. Comp. Exerc. Physiol..

[53] Dixon S.J., McNally K. (2008). Influence of orthotic devices prescribed using pressure data on lower extremity kinematics and pressures beneath the shoe during running. Clin. Biomech. Bristol Avon.

[54] Barton C.J., Menz H.B., Crossley K.M. (2011). Clinical predictors of foot orthoses efficacy in individuals with patellofemoral pain. Med. Sci. Sports Exerc..

[55] Jor A., Lau N.W.K., Daryabor A., Koh M.W.P., Lam W.-K., Hobara H., Kobayashi T. (2024). Effects of foot orthoses on running kinetics and kinematics: A systematic review and meta-analysis. Gait Posture.

[56] Lescure Y. (2017). Le syndrome fémoro-patellaire du coureur à pied. Rev. Podol..

[57] Foroughi N., Smith R., Vanwanseele B. (2009). The association of external knee adduction moment with biomechanical variables in osteoarthritis: A systematic review. Knee.

[58] Toda Y., Tsukimura N., Kato A. (2004). The effects of different elevations of laterally wedged insoles with subtalar strapping on medial compartment osteoarthritis of the knee. Arch. Phys. Med. Rehabil..

[59] Arnold J.B., Wong D.X., Jones R.K., Hill C.L., Thewlis D. (2016). Lateral Wedge Insoles for Reducing Biomechanical Risk Factors for Medial Knee Osteoarthritis Progression: A Systematic Review and Meta-Analysis. Arthritis Care Res..

[60] Radzimski A.O., Mündermann A., Sole G. (2012). Effect of footwear on the external knee adduction moment—A systematic review. Knee.

[61] Roper J.L., Harding E.M., Doerfler D., Dexter J.G., Kravitz L., Dufek J.S., Mermier C.M. (2016). The effects of gait retraining in runners with patellofemoral pain: A randomized trial. Clin. Biomech. Bristol Avon.

[62] Barton C.J., Bonanno D.R., Carr J., Neal B.S., Malliaras P., Franklyn-Miller A., Menz H.B. (2016). Running retraining to treat lower limb injuries: A mixed-methods study of current evidence synthesised with expert opinion. Br. J. Sports Med..

[63] Bonacci J., Hall M., Saunders N., Vicenzino B. (2018). Gait retraining versus foot orthoses for patellofemoral pain: A pilot randomised clinical trial. J. Sci. Med. Sport.

[64] Roosens E., Beaufils C., Busegnies Y., Van Tiggelen D. (2023). Intrinsic risk factors associated with iliotibial band syndrome: A systematic review. Spor Hekim. Derg..

